# Cancer risk in patients with nontuberculous mycobacterial pulmonary disease: a nationwide population-based cohort study

**DOI:** 10.1186/s12931-026-03811-2

**Published:** 2026-07-16

**Authors:** Chanho Lee, Hyeon Kyu Choi, Hyeok-Hee Lee, Young Ae Kang, Eun Young Kim, Hyung Woo Kim, Naeun Shin, Youngmok Park

**Affiliations:** 1 Division of Pulmonary and Critical Care Medicine, Department of Internal Medicine, Severance Hospital, Yonsei University College of Medicine, Seoul, Republic of Korea; 2 Department of Pharmacology, Yonsei University College of Medicine, Seoul, Republic of Korea; 3 Richard A. and Susan F. Smith Center for Outcomes Research, Beth Israel Deaconess Medical Center, Harvard Medical School, Boston, MA USA; 4 Department of Preventive Medicine, Yonsei University College of Medicine, Seoul, Republic of Korea; 5 Institute for Innovation in Digital Healthcare, Yonsei University, Seoul, Republic of Korea; 6 Department of Internal Medicine, Institute of Kidney Disease Research, Yonsei University College of Medicine, Seoul, Republic of Korea; 7 Yonsei University College of Medicine, Seoul, Republic of Korea

**Keywords:** Lung Neoplasms, Mycobacterium Infections, Nontuberculous, Neoplasms

## Abstract

**Background:**

Although nontuberculous mycobacterial pulmonary disease (NTM-PD) is becoming increasingly prevalent worldwide, its associated cancer risk remains largely unknown. In this study, we aimed to determine whether patients with NTM-PD are at a higher risk of developing cancer than the general population.

**Methods:**

In this retrospective nationwide cohort study using the National Health Insurance Service-National Health Information Database of South Korea, we identified 21,879 patients newly diagnosed with NTM-PD between 2010 and 2020, and included 218,790 age- and sex-matched control participants. Cumulative incidences of various cancer types were analyzed using competing risk analysis with all-cause mortality as a competing event, and cause-specific hazard ratios (HRs) were estimated using Cox proportional hazards models adjusted for demographic factors and baseline health checkup results.

**Results:**

After adjustment, patients with NTM-PD showed a significantly higher risk of overall cancer than did matched controls (adjusted HR, 1.22; 95% confidence interval, 1.17–1.27; *P* < .001). In the analysis according to cancer type, the following cancers showed significant changes in risk (adjusted HR [95% confidence interval]): lung cancer (2.25 [2.06–2.45]), multiple myeloma (1.74 [1.26–2.42]), ovarian cancer (1.33 [1.06–1.68]), pancreatic cancer (1.26 [1.09–1.45]), and liver cancer (1.22 [1.07–1.39]). By contrast, stomach (0.83 [0.72–0.96]) and colorectal (0.83 [0.73–0.95]) cancers showed a reduced risk. These associations remained robust across multiple sensitivity analyses.

**Conclusions:**

Compared with the general population, patients with NTM-PD are at an increased risk of various cancers, particularly lung cancer. These findings underscore the need for increased cancer surveillance and long-term monitoring of patients with NTM-PD.

**Supplementary Information:**

The online version contains supplementary material available at https://doi.org/10.1186/s12931-026-03811-2.

## Background

Nontuberculous mycobacterial pulmonary disease (NTM-PD) is a growing global health concern, with its incidence and prevalence steadily increasing across different populations [1, 2]. NTM species are ubiquitous opportunistic environmental pathogens that can cause chronic pulmonary infections, particularly in individuals with structural lung disease or impaired immunity [3, 4]. NTM-PD is clinically characterized by chronic respiratory symptoms, progressive radiographic deterioration, and prolonged antibiotic regimens with suboptimal outcomes [5]. It poses significant diagnostic, treatment, and long-term care challenges, particularly in aging and immunocompromised populations [3, 6].

Given the chronic inflammatory nature of NTM-PD and its impact on pulmonary architecture, its potential association with cancer, particularly lung cancer, has gained increasing interest. The prevalence of lung cancer among patients with NTM-PD is 2%–8.5%, significantly higher than that in comparable populations [7]. NTM-induced chronic inflammation and scarring may create a pro-oncogenic microenvironment, whereas lung cancer and its treatments may increase susceptibility to opportunistic NTM infections [8, 9]. The immunosuppressive tumor microenvironment and impaired host inflammatory responses associated with lung cancer may further contribute to this vulnerability[10–12]. However, the temporal directionality and causality of this association remain unclear.

Recent studies have expanded the scope of this association to include patients with extrapulmonary malignancies. Specifically, one study found that patients with NTM-PD had a significantly higher prevalence of lung cancer and other cancers (excluding breast, ovarian, and prostate cancer) than matched controls[13]. Furthermore, somatic mutations in cancer susceptibility genes such as *BRCA1*/*2* and *ERBB2* have been detected in sputum samples from women with NTM-PD, suggesting that chronic NTM infection may trigger cellular transformation and increase the oncogenic risk [14]. However, whether NTM-PD plays a causal role in carcinogenesis remains to be elucidated and data regarding cancer-specific risks in this population are limited.

The aim of this study was to investigate the association between NTM-PD and subsequent cancer development using a nationwide population-based cohort derived from the National Health Insurance Service-National Health Information Database (NHIS-NHID) of South Korea. We quantified overall and site-specific cancer risks in patients newly diagnosed with NTM-PD, compared them to those of matched controls, and identified clinical and demographic characteristics associated with an increased likelihood of malignancy.

## Methods

### Data source and ethics

We conducted a retrospective nationwide cohort study using NHIS-NHID data collected between 2002 and 2022. The NHIS is a single compulsory health insurer covering the entire Korean population. The NHIS-NHID includes comprehensive information such as demographic characteristics, insurance eligibility, inpatient and outpatient medical claims with diagnostic codes (based on the International Classification of Disease, 10th Revision [ICD-10]), prescription records, and biennial health examination results. The standardized health screening program, offered to all insured adults aged ≥ 40 years since 1995, captures anthropometric measurements and lifestyle questionnaires covering smoking patterns, alcohol use, and physical activity. The overall structure and validity of the NHIS-NHID have been described previously [15].

This study was conducted in accordance with the Declaration of Helsinki and was approved by the Institutional Review Board of Severance Hospital (4–2024-1640) and the Korea NHIS Medical Information Disclosure Committee (NHIS-2024–1–250). Given the retrospective nature of this study and the use of de-identified administrative data, the requirement for informed consent was waived. This study was reported in accordance with the Strengthening the Reporting of Observational Studies in Epidemiology reporting guidelines.

### Study population

The exposed cohort comprised adults aged 40–90 years with NTM-PD that was newly diagnosed between January 1, 2010 and December 31, 2020. The operational definition of NTM-PD identification required: (1) ≥ 2 medical claims from outpatient or inpatient visits with NTM diagnostic codes (ICD-10: A31.0, A31.8, A31.9) within 180 days and (2) ≥ 1 acid-fast bacilli culture claim within 180 days preceding diagnosis. The requirement for repeated claims within a 180-day window was intended to identify active clinical episodes of NTM-PD while excluding single incidental or rule-out diagnoses [16, 17]. The 180-day culture window reflects clinical practice by accounting for the prolonged incubation period (up to 8 weeks) and subsequent species identification required for mycobacterial diagnosis, while ensuring temporal linkage between microbiological testing and clinical diagnosis. The exclusion criteria were individuals with pre-existing malignancy before NTM-PD diagnosis to ensure incident cancer assessment, age < 40 or > 90 years (outside of health screening eligibility range), and absence of health examination data within 1 year before diagnosis to ensure complete baseline covariate measurements. The index date corresponded to the NTM-PD diagnosis date, with observations continuing until cancer occurrence, death, or the end of database availability (December 31, 2022).

Unexposed control participants without a history of NTM diagnosis were matched 1:10 by age and sex to patients with NTM-PD sharing the same index date. The same exclusion criteria for patients with NTM-PD (no prior malignancy or available health examination data) were also applied to control participants at the index date during matching.

### Outcomes and covariables

The primary outcome was incident cancer, defined as the first occurrence of any malignancy during the follow-up period, based on ICD-10 diagnostic codes. Secondary outcomes included site-specific cancers, categorized according to primary anatomical location: lung (C34), stomach (C16), colorectum (C18–C21), liver (C22), biliary tract (C23–C24), pancreatic (C25), thyroid (C73), kidney (C64–C65), bladder (C67), head and neck (C00–C14, C30–C32), breast (C50), ovary (C56), prostate (C61), male genital organs other than the prostate (C60, C62–C63), cervix (C53), female genital organs other than the cervix and ovary (C51–C52, C54–C55, C57–C58), multiple myeloma (C90), leukemia (C91–C95), and lymphoma (C81–C85).

**Fig. 1:**
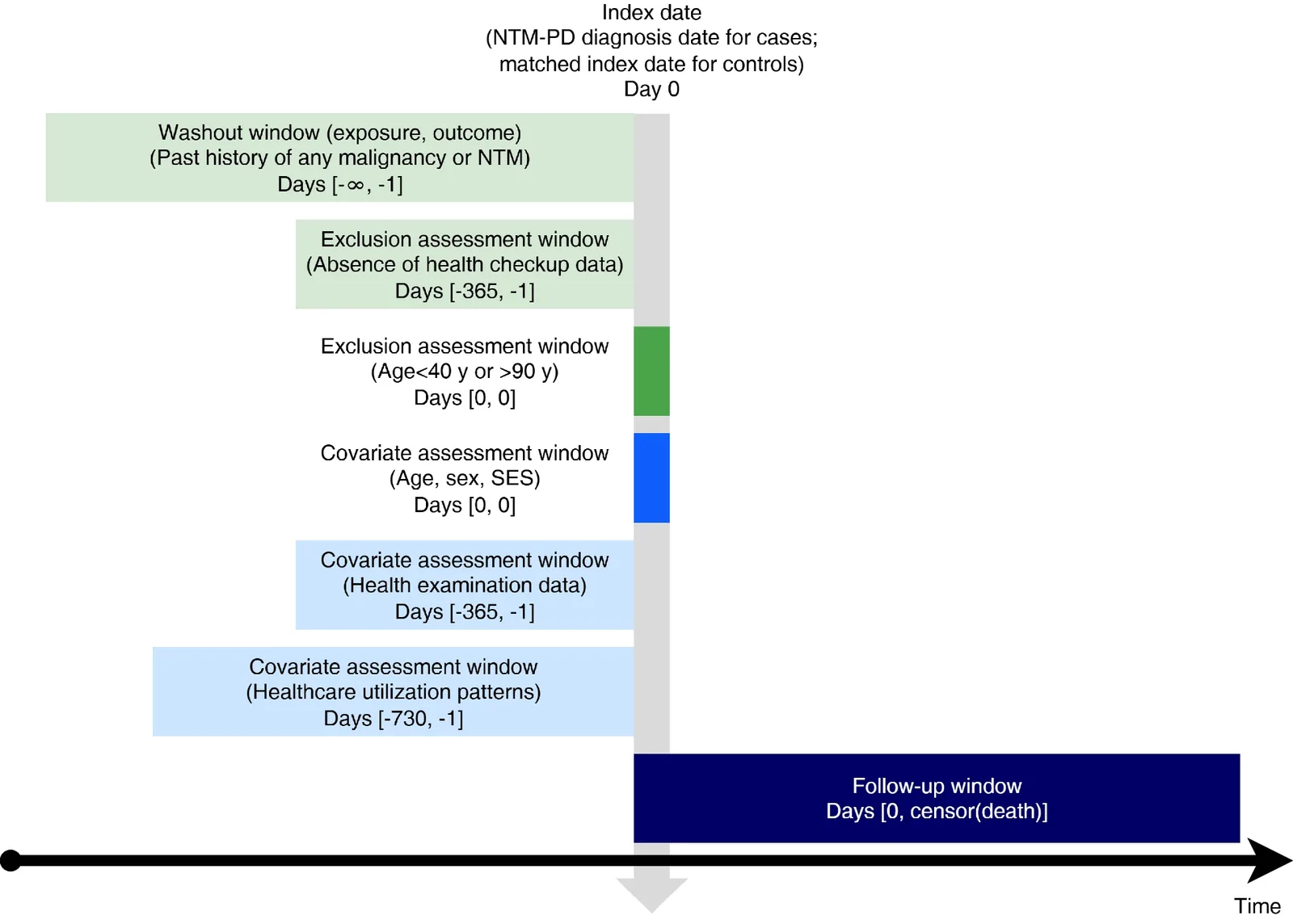
Timeline of assessment windows and temporal anchors in the study design. Control participants were matched 1:10 by age and sex with patients with NTM-PD sharing the same index date as the corresponding matched patient. Baseline covariates were assessed during specified windows relative to the index date (as detailed in the Methods section). Follow-up continued from the index date until cancer occurrence, death, or the end of the study period (December 31, 2022). NTM-PD, nontuberculous mycobacterial pulmonary disease; SES, socioeconomic status

Baseline demographic characteristics included age, sex, residential area type (metropolitan, urban, or rural), and insurance premium quintile as proxies for socioeconomic status. Health behaviors and anthropometric measurements were obtained from national health screening examinations, including tobacco smoking status (current smoker, ex-smoker, never smoker), physical activity levels (frequency of moderate or vigorous intensity exercise or physical inactivity), alcohol consumption patterns (frequency of alcohol consumption or “non-drinker” status), and body mass index (BMI; underweight [< 18.5 kg/m^2^], normal [18.5–25 kg/m^2^], or overweight/obese [≥ 25 kg/m^2^]). Healthcare utilization patterns were quantified by the total number of outpatient visits and chest computed tomography (CT) scans during the 2 years before the index date. The timeline of the assessment windows and temporal anchors relative to the index date is presented in Fig. 1 [18].

### Statistical analysis

Continuous variables are presented as means with standard deviations or medians with interquartile ranges, while categorical variables as absolute numbers with percentages. Intergroup differences were compared using the Student’s t-test and chi-squared test. To visualize temporal trends in incidence rates, the cumulative incidence of cancer in both groups was estimated using competing risk analysis with all-cause mortality as a competing event, employing the Fine–Gray subdistribution hazard model [19].

To compare cancer risks between patients with NTM-PD and control participants, cause-specific hazard ratios (HRs) with 95% confidence intervals (CIs) for incident cancer and site-specific cancer types were calculated using Cox proportional hazards models. The main model was adjusted for age, sex, residential area type, insurance premium quintile, tobacco smoking status, BMI, exercise frequency, and alcohol consumption frequency. To address potential false discovery risks in the main model from multiple hypotheses in different types of cancers, *P*-values were adjusted for multiple comparisons with a false discovery rate (FDR) of 0.05, using the MULTTEST procedure in SAS software (SAS Institute, Cary, NC, USA) based on the Benjamini–Hochberg method [20, 21]. The FDR adjustment was applied across all 20 hypotheses tested, encompassing overall cancer and 19 site-specific cancer types.

Sensitivity analyses were performed with additional adjustments: (1) including total outpatient visits during the 2 years before the index date to adjust for healthcare utilization bias; (2) excluding participants who developed cancer within the first year after the index date to address potential detection bias; and (3) censoring at the time of diagnosis of other cancer types to focus on site-specific risks. For lung cancer specifically, an additional sensitivity analysis was conducted with adjustment for prior chest CT scans because the diagnostic workup for NTM-PD may result in incidental lung cancer detection.

As post-hoc sensitivity analyses, we performed two additional analyses to address potential temporal and treatment-related confounding. First, given that the study period extended through 2022, we could not rule out the influence of the COVID-19 pandemic on cancer diagnosis and healthcare utilization. We therefore repeated the main analysis with follow-up censored on December 31, 2019. Second, to examine whether cancer risk varied by treatment status among patients with NTM-PD, we performed a time-varying Cox regression to address potential immortal time bias [22]. Since *Mycobacterium avium* complex and *Mycobacterium abscessus* account for approximately 94% of NTM-PD cases in Korea, macrolides—clarithromycin and azithromycin—form the backbone of NTM-PD treatment regimens [23, 24]. The primary treatment-stratified analysis used cumulative combined macrolide exposure (clarithromycin and/or azithromycin) of ≥ 365 days as the threshold, reflecting the minimum recommended duration of guideline-based therapy. Patients contributed person-time as untreated from the index date until reaching this cumulative threshold and as treated thereafter. We additionally performed parallel analyses using a shorter exposure threshold (≥ 180 days) and analyzing clarithromycin and azithromycin separately to assess the robustness of the primary findings. Other antibiotics commonly used in NTM-PD treatment could not be analyzed because the NHIS-NHID does not release prescription records for agents supplied by a single manufacturer, owing to the risk of patient re-identification.

Subgroup analyses were conducted for all cancer types by stratifying patients according to sex (male/female), age (< 65/≥ 65 years), BMI category (< 18.5/18.5–25/≥ 25 kg/m^2^), smoking status (current/ex/never), exercise status (yes/no), and alcohol consumption (yes/no). *P*-values for interactions were calculated to assess the potential effect modifications. An additional sensitivity analysis was conducted for the subgroup findings using the Fine–Gray subdistribution hazard model, rather than the cause-specific hazard model, to account for competing mortality risk.

Two-sided *P*-values < 0.05 were considered statistically significant. All statistical analyses were performed using SAS version 9.4 (SAS Institute). Graphical representations of the data were generated using R version 4.3.1 (R Foundation for Statistical Computing, Vienna, Austria).

## Results

### Baseline characteristics

**Fig. 2:**
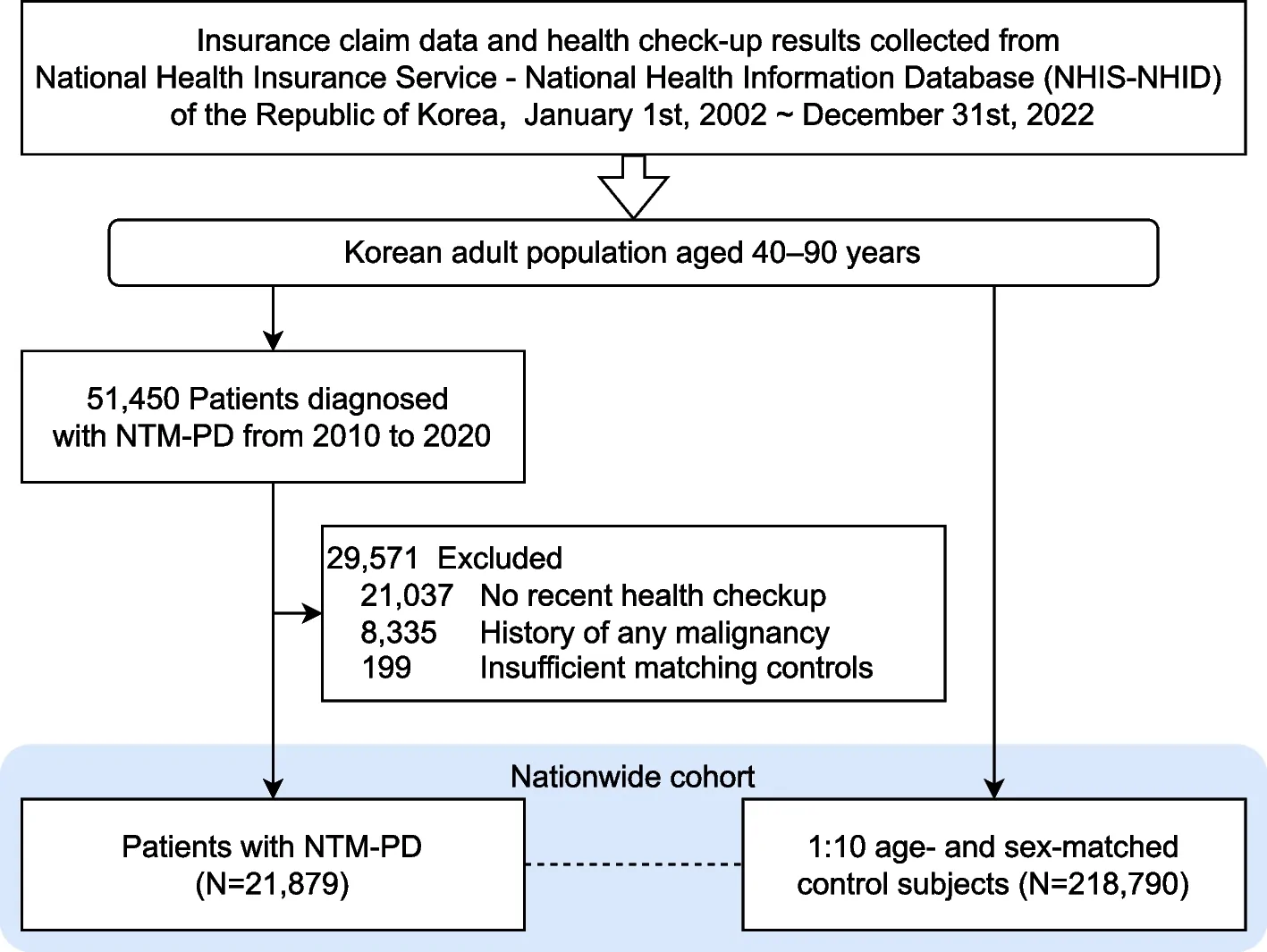
Flow diagram of the study population selection and matching process. The inclusion and exclusion criteria were sequentially applied for patients with NTM-PD and matched control participants. Control participants were randomly sampled from the eligible population during matching, with identical exclusion criteria applied; exclusion counts for control participants are not displayed because of the sampling method. NTM-PD, nontuberculous mycobacterial pulmonary disease

The nationwide cohort initially included 51,450 patients newly diagnosed with NTM-PD between January 1, 2010 and December 31, 2020. Patients who did not undergo national health screening in the year of or year prior to diagnosis (*n* = 21,037) were excluded. Patients with a history of cancer pre-NTM-PD diagnosis (*n* = 8,335) were also excluded. Finally, 199 patients who could not be age- and sex-matched were excluded. After applying these criteria, 21,879 patients with NTM-PD were included in the final cohort (Fig. 2). For the control group, 218,790 individuals were selected through 1:10 age and sex matching with the NTM-PD cohort.

In both groups, the mean age was 63.5 years, with 46.5% of participants aged ≥ 65 years (Table 1). The proportion of men was also the same in both groups (40.3%). The median follow-up duration was significantly shorter in the NTM-PD group than in the control group (5.5 vs. 5.9 years, *P* < 0.001), while the all-cause mortality during the follow-up period was higher (14.4% vs. 7.6%, *P* < 0.001).

**Table 1:** Baseline characteristics of the study population

**Characteristic**		**Patients with** **NTM-PD**	**Control participants**	
		(*n* = 21,879)	(*n* = 218,790)	*P*
Age, years	Mean (SD)	63.5 (10.7)	63.5 (10.7)	>.99
≥ 65	n (%)	10,180 (46.5)	101,800 (46.5)	>.99
Male sex	n (%)	8,822 (40.3)	88,220 (40.3)	>.99
Follow-up, years	Median (IQR)	5.5 (3.5–8.2)	5.9 (3.8–8.5)	<.001
All-cause mortality during follow-up	n (%)	3,179 (14.4)	16,735 (7.6)	<.001
Area				<.001
Metropolitan	n (%)	9,757 (44.6)	94,354 (43.1)	
Urban	n (%)	9,787 (44.7)	100,997 (46.2)	
Rural	n (%)	2,335 (10.7)	23,439 (10.7)	
Insurance contributions quintile				<.001
Highest 20%	n (%)	7,581 (34.7)	64,983 (29.7)	
20%–40%	n (%)	4,435 (20.3)	43,717 (20.0)	
40%–60%	n (%)	3,094 (14.1)	33,897 (15.5)	
60%–80%	n (%)	2,640 (12.1)	31,401 (14.4)	
Lowest 20%	n (%)	4,129 (18.9)	44,792 (20.5)	
Baseline health at checkup				
Smoking status				<.001
Current smoker	n (%)	2,392 (10.9)	34,337 (15.7)	
Ex-smoker	n (%)	3,234 (14.8)	28,895 (13.2)	
Never smoker	n (%)	16,253 (74.3)	155,558 (71.1)	
Exercise, times/week				
Moderate intensity	Mean (SD)	1.5 (2.1)	1.6 (2.1)	.006
Vigorous intensity	Mean (SD)	0.9 (1.7)	1.0 (1.8)	<.001
Physically inactive	n (%)	10,454 (47.8)	101,838 (46.6)	<.001
Alcohol intake, times/month	Mean (SD)	2.2 (5.6)	3.9 (6.7)	<.001
Non-drinker	n (%)	16,189 (74.0)	106,957 (48.9)	<.001
BMI, kg/m^2^	Mean (SD)	21.4 (3.0)	24.1 (3.2)	<.001
< 18.5	n (%)	3,361 (15.4)	5,214 (2.4)	<.001
18.5–25	n (%)	15,933 (72.8)	133,474 (61.0)	
≥ 25	n (%)	2,585 (11.8)	80,102 (36.6)	
Baseline healthcare utilization				
OPD visits, per 2 years	Mean (SD)	63.2 (54.5)	45.7 (46.3)	<.001
Chest CT scans, per 2 years	Mean (SD)	1.6 (1.4)	0.1 (0.4)	<.001

*BMI* body mass index, *CT* computed tomography, *IQR* interquartile range, *NTM-PD* nontuberculous mycobacterial pulmonary disease, *OPD* outpatient department, *SD* standard deviation

At the baseline health checkup, current smokers were less common in the NTM-PD group than in the control group (10.9% vs. 15.7%), whereas the proportion of ex-smokers was slightly higher (14.8% vs. 13.2%, *P* < 0.001). Patients with NTM-PD reported lower levels of moderate and vigorous physical activity and less frequent alcohol consumption than the control participants (all *P* < 0.001). The mean BMI was significantly lower in the NTM-PD group than in the control group (21.4 vs. 24.1 kg/m^2^, *P* < 0.001). Underweight (BMI < 18.5 kg/m^2^) was substantially more prevalent in the NTM-PD group (15.4% vs. 2.4%), while obesity (BMI ≥ 25 kg/m^2^) was less common (11.8% vs. 36.6%). Regarding healthcare utilization, the number of outpatient department visits and chest CT scans was higher in the NTM-PD group than in the control group (63.2 vs. 45.7, *P* < 0.001 and 1.6 vs. 0.1, *P* < 0.001, respectively).

### Cumulative cancer risk in NTM-PD

**Fig. 3:**
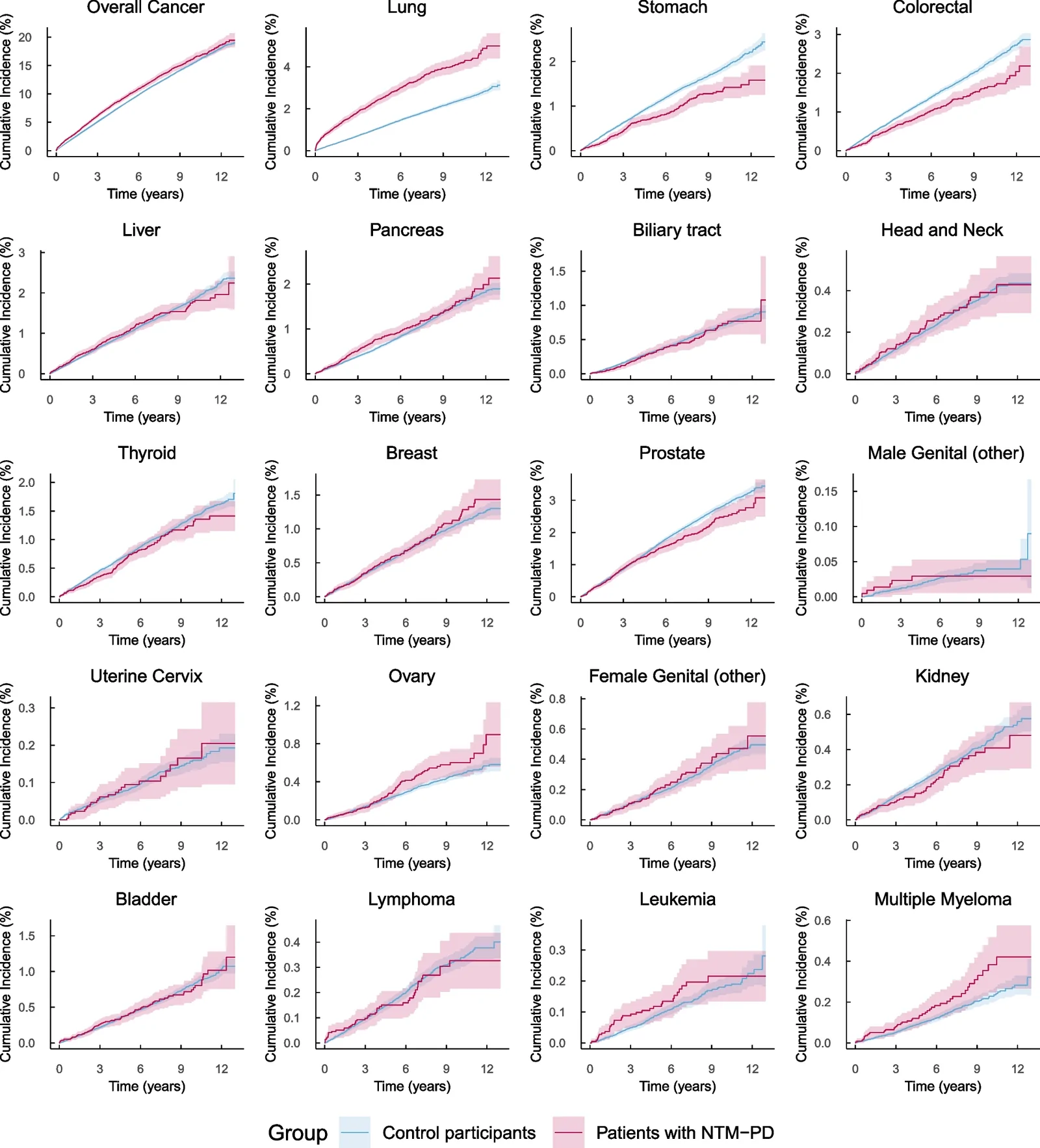
Cumulative incidence of overall and site-specific cancer in patients with NTM-PD and matched control participants. Cumulative incidences were estimated using the Fine–Gray subdistribution hazard model, with all-cause mortality as a competing event. The solid lines represent the cumulative incidence curves and the shaded areas denote 95% confidence intervals. NTM-PD, nontuberculous mycobacterial pulmonary disease

Cumulative incidences using the Fine–Gray subdistribution hazard model illustrated a difference in cancer risk in patients with NTM-PD and matched control participants (Fig. 3). The divergence of the curves was the most pronounced for lung, stomach, and colorectal cancers, followed by multiple myeloma and ovarian cancer. Patients with NTM-PD had a higher cumulative incidence of lung cancer, multiple myeloma, and ovarian cancer and a lower incidence of stomach and colorectal cancers. The overall cancer incidence was higher in the NTM-PD group throughout the observation period.

**Fig. 4:**
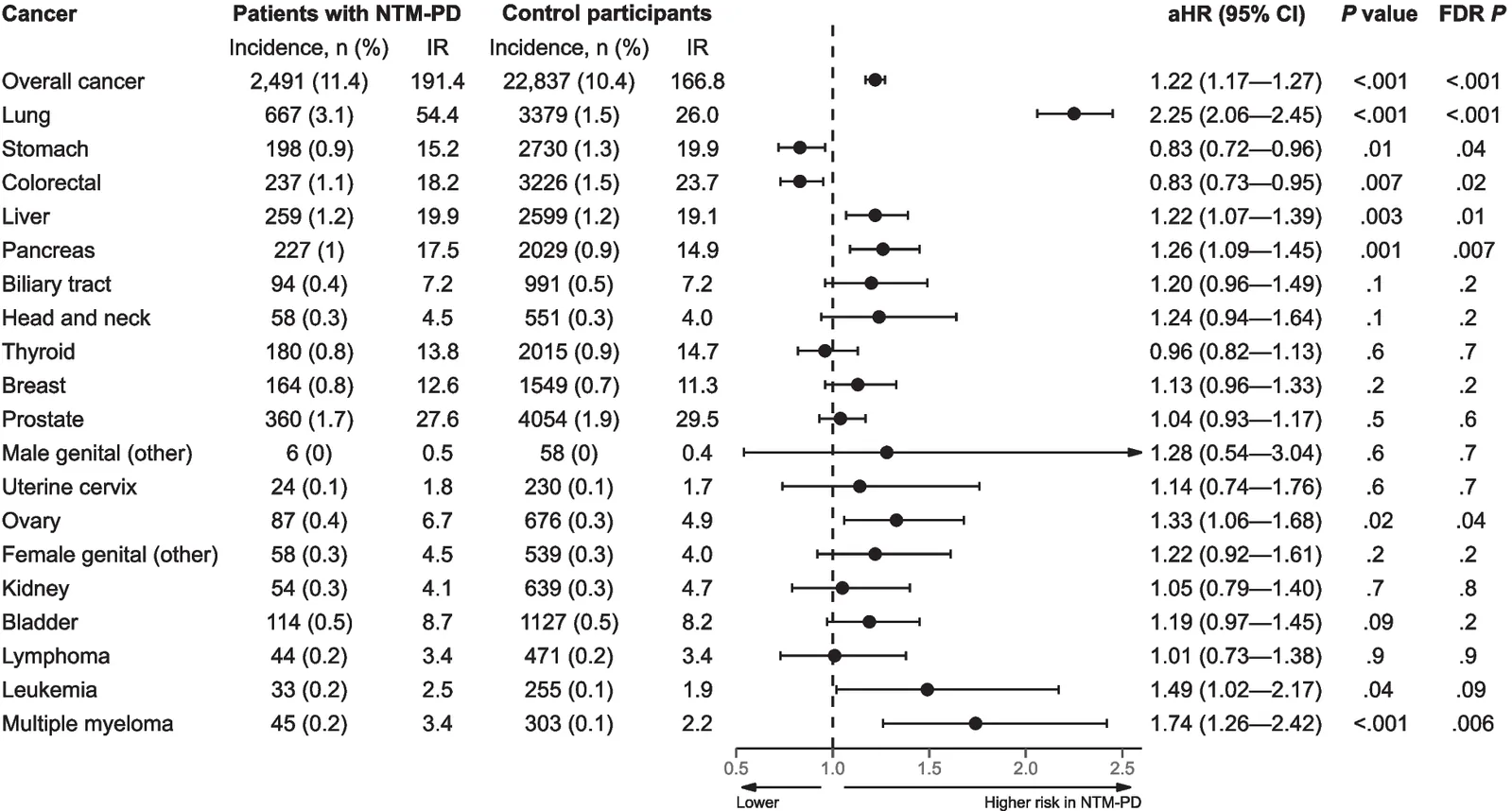
Cancer risk in patients with NTM-PD compared with those in matched control participants. Hazard ratios were calculated using Cox proportional hazards models adjusted for age, sex, residential area, socioeconomic status, and health behaviors (as detailed in the Methods section). The incidence rates were calculated per 10,000 person-years. *P*-values were adjusted for multiple comparisons using the Benjamini–Hochberg method, and statistical significance was indicated at an FDR-corrected *P* <.05. aHR, adjusted hazard ratio; CI, confidence interval; FDR, false discovery rate; IR, incidence rate; NTM-PD, nontuberculous mycobacterial pulmonary disease

Patients with NTM-PD exhibited a significantly higher incidence of cancer than age- and sex-matched control participants (11.4% vs. 10.4%; 191.4 vs. 166.8 per 10,000 person-years), with an adjusted HR (aHR) of 1.22 (95% CI, 1.17–1.27; FDR *P* < 0.001; Fig. 4). Among site-specific cancers, the most pronounced increase was observed in lung cancer. Patients with NTM-PD showed a more than two-fold higher incidence of lung cancer than matched control participants (3.1% vs. 1.5%; 54.4 vs. 26.0 per 10,000 person-years; aHR, 2.25; 95% CI, 2.06–2.45; FDR *P* < 0.001). Significantly increased risks were also observed for multiple myeloma (aHR, 1.74; 95% CI, 1.26–2.42; FDR *P* = 0.006) and ovarian (aHR, 1.33; 95% CI, 1.06–1.68; FDR *P* = 0.04), liver (aHR, 1.22; 95% CI, 1.07–1.39; FDR *P* = 0.01) and pancreatic cancers (aHR, 1.26; 95% CI, 1.09–1.45; FDR *P* = 0.007). By contrast, patients with NTM-PD had a lower risk of stomach and colorectal cancers than did control participants (0.9% vs. 1.3%; aHR, 0.83; 95% CI, 0.72–0.96; FDR *P* = 0.04 and 1.1% vs. 1.5%; aHR, 0.83; 95% CI, 0.73–0.95; FDR *P* = 0.02, respectively). There were no significant differences in the risks of other malignancies between groups.

### Sensitivity analyses

Sensitivity analyses were performed to further support the robustness of the primary analysis (Table 2). First, we adjusted for yearly outpatient visits before the index date to address healthcare utilization bias. Applying this additional confounder to the main model did not result in any significant differences in the calculated cancer risks of patients with NTM-PD. Second, participants who developed cancer within the first year were excluded. Although this only slightly changed the cancers with increased risk, the lower risks of stomach and colorectal cancers became non-significant. Nevertheless, further analysis with different time frames (2 and 3 years) showed a significantly lower risk of colorectal cancer in patients with NTM-PD (Supplementary Table 1 in Additional File 1). By contrast, the increased risks of pancreatic and liver cancers were attenuated with longer exclusion periods. Third, to focus on site-specific risks, we censored participants when other types of cancers were diagnosed. This similarly did not make any difference to the main findings of the primary model.

**Table 2:** Sensitivity analyses of the risks of incident cancer in patients with NTM-PD

	**Main Model**		**Sensitivity 1**		**Sensitivity 2**		**Sensitivity 3**		**Sensitivity 4**	
**Cancer Type**	**aHR (95% CI)**	***P***	**aHR (95% CI)**	***P***	**aHR (95% CI)**	***P***	**aHR (95% CI)**	***P***	**aHR (95% CI)**	***P***
Overall cancer	1.22 (1.17–1.27)	<.001	1.19 (1.14–1.24)	<.001	1.19 (1.13–1.25)	<.001	N/A		1.28 (1.21–1.35)	<.001
Lung	2.25 (2.06–2.45)	<.001	2.19 (2.00–2.39)	<.001	1.92 (1.74–2.13)	<.001	2.25 (2.06–2.45)	<.001	2.55 (2.28–2.84)	<.001
Stomach	0.83 (0.72–0.96)	.01	0.83 (0.72–0.97)	.02	0.89 (0.76–1.05)	.2	0.84 (0.73–0.98)	.02	0.78 (0.64–0.95)	.01
Colorectal	0.83 (0.73–0.95)	.007	0.82 (0.72–0.94)	.005	0.89 (0.77–1.03)	.1	0.84 (0.74–0.97)	.01	0.73 (0.61–0.88)	.001
Liver	1.22 (1.07–1.39)	.003	1.16 (1.02–1.32)	.03	1.20 (1.03–1.38)	.02	1.23 (1.08–1.40)	.002	1.37 (1.17–1.71)	<.001
Pancreatic	1.26 (1.09–1.45)	.001	1.19 (1.03–1.37)	.02	1.27 (1.09–1.48)	.002	1.27 (1.11–1.47)	<.001	1.42 (1.18–1.71)	<.001
Ovarian	1.33 (1.06–1.68)	.02	1.29 (1.02–1.63)	.03	1.36 (1.06–1.74)	.01	1.35 (1.07–1.70)	.01	1.16 (0.84–1.60)	.4
Multiple myeloma	1.74 (1.26–2.42)	<.001	1.67 (1.20–2.32)	.002	1.60 (1.11–2.31)	.01	1.77 (1.28–2.45)	<.001	1.80 (1.16–2.81)	.009

Sensitivity analyses using the Cox proportional hazards model from the main analysis were performed as follows: Sensitivity 1 (further adjusting for outpatient visits during the 2 years before the index date), Sensitivity 2 (excluding individuals with a short follow-up period [< 365 days]), Sensitivity 3 (censoring at the time of other cancer type diagnosis), and Sensitivity 4 (censoring follow-up on December 31, 2019 to exclude potential influence of the COVID-19 pandemic)

*aHR* Adjusted hazard ratio, *CI* Confidence interval, *N/A* Not applicable

As additional post-hoc sensitivity analyses, we further examined the potential influence of the COVID-19 pandemic and macrolide treatment. By censoring follow-up at the end of 2019, most cancer risks associated with NTM-PD remained unchanged, except for ovarian cancer, which became non-significant (Table 2).

### Lung cancer-specific analyses

An additional sensitivity analysis for lung cancer was conducted with adjustment for prior chest CT scans to account for detection bias, given that the diagnostic workup for NTM-PD may result in incidental lung cancer detection. The frequency of chest CT scans was substantially higher in patients with NTM-PD, potentially creating collinearity with NTM-PD exposure and attenuating the observed effect. Nevertheless, after additional adjustment for chest CT scans, the lung cancer risk remained elevated (HR 1.70; 95% CI 1.52–1.89; *P* < 0.001). This suggests that the association between NTM-PD and lung cancer remains robust even after accounting for increased radiological surveillance.

**Fig. 5:**
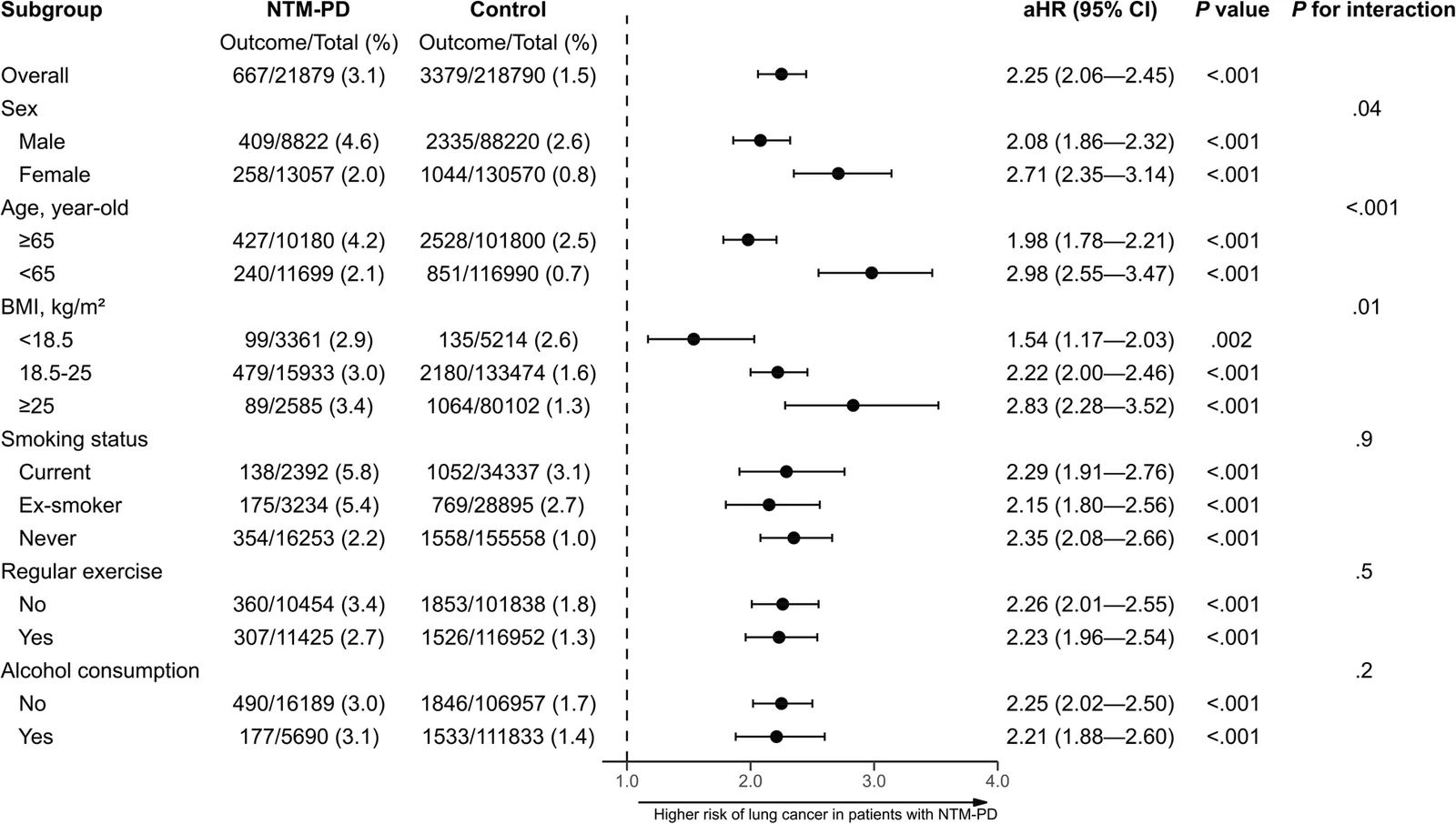
Subgroup analysis of lung cancer risk in patients with NTM-PD. Adjusted hazard ratios and 95% confidence intervals for lung cancer development in patients with NTM-PD stratified by demographic and clinical characteristics; *P-*values for interaction were calculated to assess potential effect modification across the subgroups. aHR, adjusted hazard ratio; BMI, body mass index; CI, confidence interval; NTM-PD, nontuberculous mycobacterial pulmonary disease

Subgroup analyses were performed across all cancer types to explore potential effect modifiers in the relationship between NTM-PD and cancer risk. Significant effect modification was observed only for lung cancer; no meaningful subgroup differences were detected for other cancer types. The association between NTM-PD and an increased lung cancer risk remained significant across all subgroups; however, the magnitude of this association differed. Significant interactions were observed among subgroups according to sex, age, and BMI (Fig. 5). Notably, the association between NTM-PD and lung cancer was attenuated in men (HR 2.08 vs. 2.71 in women, *P*_interactio*n*_ = 0.04), patients aged ≥ 65 years (HR 1.98 vs. 2.98 in < 65 years, *P*_interaction_ < 0.001), and patients with a BMI < 18.5 kg/m^2^ (HR 1.54 vs. 2.83 in BMI ≥ 25 kg/m^2^, *P*_interactio*n*_ = 0.01). In contrast, the increased lung cancer risk in patients with NTM-PD was observed regardless of smoking status (*P*_interaction_ = 0.9), indicating that NTM-PD contributes to lung cancer risk independently of smoking. Given that male sex, older age, and low BMI are established mortality risk factors in patients with NTM-PD, the attenuated associations observed in these subgroups could reflect competing mortality rather than a true reduction in lung cancer risk. To address this, we repeated the subgroup analyses using the Fine–Gray subdistribution hazard model to account for competing mortality. The subgroup-specific patterns remained consistent with the main analysis (Supplementary Fig. 1 in Additional File 1).

### Macrolide treatment within the NTM-PD subcohort

Within the NTM-PD subcohort, time-varying Cox regression was used to assess whether cancer risk differed by macrolide treatment status. In the primary analysis, cumulative combined macrolide exposure (clarithromycin and/or azithromycin) of ≥ 365 days was associated with a higher overall cancer risk relative to untreated person-time (HR 1.21, 95% CI 1.09–1.34, *P* < 0.001; Supplementary Table 2 in Additional File 1). The most pronounced association was observed for lung cancer (HR 1.56, 95% CI 1.27–1.91, *P* < 0.001). The elevated lung cancer risk was consistently observed across all parallel analyses, including a shorter exposure threshold (≥ 180 days) and separate analyses of clarithromycin and azithromycin, reaching statistical significance in most analyses (Supplementary Tables 2–3 in Additional File 1). Findings for overall cancer were more variable. Azithromycin was associated with elevated risk at both thresholds, while clarithromycin showed no significant association at either threshold.

## Discussion

This study investigated the association between NTM-PD and subsequent cancer development using a nationwide population-based cohort in South Korea. Patients with NTM-PD had a significantly higher risk of cancer than age- and sex-matched control participants. This elevated risk was most pronounced for lung cancer, with a more than twofold higher incidence, followed by multiple myeloma and ovarian, pancreatic, and liver cancers. However, the incidence of stomach and colorectal cancers was lower in the NTM-PD group than in the control group, suggesting site-specific heterogeneity in the cancer risk. These findings remained robust across multiple sensitivity analyses. Subgroup analyses revealed that female sex, younger age (< 65 years), and higher BMI (≥ 25 kg/m^2^) were associated with stronger associations between NTM-PD and lung cancer risk. These findings provide robust epidemiological evidence to guide future cancer surveillance strategies and inform integrated management approaches for patients with NTM-PD.

The increased risk of lung cancer among these patients is consistent with previous observational studies. This association may reflect shared clinical features, radiographic similarities, and overlapping risk factors such as smoking and chronic inflammation [8, 25]. Chronic pulmonary inflammation induced by persistent NTM infection may create a microenvironment conducive to carcinogenesis through repeated epithelial injury, cytokine release, and altered immune surveillance [7, 8]. These processes mirror the mechanisms observed in tuberculosis-related lung cancer risk. Consistent with this hypothesis, our treatment-stratified analysis showed cumulative macrolide therapy of ≥ 365 days was associated with elevated lung cancer risk within the NTM-PD cohort, with similar associations observed across shorter exposure thresholds and individual macrolide analyses. Because the NHIS-NHID does not capture direct measures of NTM-PD severity, prolonged macrolide use likely reflects more clinically significant or progressive NTM-PD, supporting the notion that greater cumulative inflammatory burden corresponds to greater cancer risk.

Furthermore, the elevated incidence of hematologic malignancies, particularly multiple myeloma, may reflect immune dysregulation related to T-cell exhaustion in NTM-PD [26, 27]. NTM infections trigger Th1/Th17-mediated immune responses and cytokine cascades, which have been implicated in plasma cell dyscrasia and other hematological cancers. The increased risks of ovarian and pancreatic cancer observed here are consistent with a recent report suggesting a predisposition in patients with NTM-PD to mutations in oncogenic genes such as *BRCA1*/*2* [14]. Beyond genetic predisposition, impaired immune surveillance may also contribute. Chronic NTM infection is associated with T-cell exhaustion and sustained immune dysregulation, which may weaken tumor immune surveillance and permit tumorigenesis [27, 28]. This mechanism may act together with the underlying genetic susceptibility to increase the risk of ovarian and pancreatic cancer.

Our findings also revealed significantly lower incidences of stomach and colorectal cancers in the NTM-PD group. This inverse association could be attributed to differences in healthcare utilization patterns, such as more frequent medical visits or enhanced surveillance, potentially leading to earlier detection and prevention. However, this lower incidence persisted even after adjusting for healthcare utilization patterns in our sensitivity analysis, suggesting that the observed lower risks were not merely due to differences in medical care access or surveillance intensity. Therefore, NTM-PD may exert organ-specific and differential effects on carcinogenesis. This is further supported by the lack of association observed for several other cancer types, including prostate, thyroid, and kidney cancers, underscoring that NTM-PD does not confer a uniform oncogenic risk across all tissues. Collectively, these results suggest complex and tissue-specific immune-tumor interactions in the context of NTM-PD. However, further studies are required to validate these findings.

Subgroup analyses further demonstrated that the association between NTM-PD and lung cancer risk remained consistent across different demographic and clinical subgroups, although the association magnitude varied significantly according to sex, age, and BMI. Notably, subgroups with attenuated associations (male sex, older age, and low BMI) corresponded to the high-risk components of the BACES score, which is used to predict mortality in patients with NTM-PD [29]. This pattern may suggest that competing mortality risks may reduce the incidence of lung cancer in vulnerable populations. However, our competing-risk analysis using the Fine–Gray subdistribution hazard model confirmed that subgroup differences persisted even after accounting for differential mortality rates. These findings have important clinical implications. Female sex, younger age, and obesity are generally considered protective factors against lung cancer in the general population [30, 31]. Therefore, patients with NTM-PD with these traditionally lower-risk characteristics may require heightened surveillance for lung cancer development.

This study had several strengths. By using the NHIS-NHID, which covers the entire Korean population, we achieved large-scale representative sampling and robust statistical power. Some smaller prior studies did not detect significant associations between NTM-PD and certain cancer types, likely reflecting limited statistical power and shorter follow-up [13, 14]. The present nationwide cohort, with its substantially larger sample size and extended follow-up, was better positioned to detect modest but clinically relevant associations. We applied rigorous methodological approaches, including excluding cases with prior cancer, careful time-alignment of exposures and outcomes, competing risk analysis to account for elevated mortality in patients with NTM-PD, and multivariate adjustment to minimize confounding. Extensive sensitivity analyses mitigated various potential biases, including adjustments for healthcare utilization patterns, exclusion of patients with cancer diagnosed within the first year to address detection bias, and site-specific censoring approaches to focus on individual cancer risk. Cancer outcomes were comprehensively assessed through longitudinal follow-up, and we adjusted for key behavioral and metabolic factors derived from national health screening data. Furthermore, examining site-specific cancers individually provided a nuanced understanding of the differential risks associated with NTM-PD.

This study also had some limitations. First, the observational design of this study limits the ability to draw definitive causal inferences. Second, claims-based diagnostic codes may have introduced misclassification bias. However, we used insurance claims for NTM-PD microbiological testing to enhance diagnostic specificity. For cancer outcomes, diagnostic validity has been strengthened by the Special Calculation Exceptions program of South Korea, which incentivizes accurate cancer registration through comprehensive reimbursement coverage. Third, the study lacked data on certain clinical variables such as radiographic patterns, bacterial loads, and inflammatory markers, which may influence the risk of cancer or serve as proxies for the severity of NTM-PD. This limitation also applies to the treatment-stratified analysis, where residual confounding by indication cannot be fully excluded. Fourth, the claims data did not allow histologic classification of lung cancer into adenocarcinoma, squamous cell, large cell, and small cell subtypes. Because these subtypes differ in their relationship with smoking and chronic inflammation, subtype-resolved analysis could clarify the mechanisms underlying the increased lung cancer risk in NTM-PD and warrants investigation in future studies[32, 33]. Fifth, although BMI was included as a continuous covariate, residual confounding factors due to unmeasured lifestyle or genetic factors could not be fully excluded. Finally, we cannot fully exclude the influence of the COVID-19 pandemic on our findings. Patients with NTM-PD have been reported to experience a higher incidence of COVID-19 than the general population, which may have led to differential pandemic-related healthcare disruptions between the cohorts [34, 35]. The loss of statistical significance in ovarian cancer risk in the pre-pandemic analysis suggests that this borderline association may have been influenced by pandemic-related shifts in healthcare utilization and cancer diagnosis patterns [35]. Nevertheless, the sensitivity analysis indicated that the remaining findings were robust.

## Conclusions

When compared to the general population, patients with NTM-PD had a higher risk of developing cancer, particularly lung, ovarian, liver, and pancreatic cancers and multiple myeloma. These findings highlight the importance of cancer surveillance in patients with NTM-PD. Clinicians should consider comprehensive screening strategies and maintain a high suspicion of cancer in this population.

## Supplementary Information

Supplementary Material 1.

## Data Availability

Data are publicly available.

## Abbreviations

NTM-PD: Nontuberculous mycobacterial pulmonary disease
NHIS-NHID: National Health Insurance Service-National Health Information Database
ICD-10: International Classification of Disease, 10th Revision
BMI: Body mass index
HR: Hazard ratio
CI: Confidence interval
FDR: False discovery rate
aHR: Adjusted hazard ratio

## References

[1] Prevots DR, Marshall JE, Wagner D, Morimoto K. Global epidemiology of nontuberculous mycobacterial pulmonary disease: a review. Clin Chest Med. 2023;44:675–721. 10.1016/j.ccm.2023.08.012 PMC10625169 37890910

[2] Kim JY, Kwak N, Yim JJ. The rise in prevalence and related costs of nontuberculous mycobacterial diseases in South Korea, 2010-2021. Open Forum Infect Dis. 2022;9:ofac649. 10.1093/ofid/ofac649 PMC9772866 36570964

[3] Henkle E, Winthrop KL. Nontuberculous mycobacteria infections in immunosuppressed hosts. Clin Chest Med. 2015;36:91–9. 10.1016/j.ccm.2014.11.002 PMC4710582 25676522

[4] Jeon D. Infection source and epidemiology of nontuberculous mycobacterial lung disease. Tuberc Respir Dis (Seoul). 2019;82:94–101. 10.4046/trd.2018.0026 PMC6435933 30302953

[5] Tortoli E. Clinical manifestations of nontuberculous mycobacteria infections. Clin Microbiol Infect. 2009;15:906–10. 10.1111/j.1469-0691.2009.03014.x 19845702

[6] Mirsaeidi M, Farshidpour M, Ebrahimi G, Aliberti S, Falkinham JO 3rd. Management of nontuberculous mycobacterial infection in the elderly. Eur J Intern Med. 2014;25:356–63. 10.1016/j.ejim.2014.03.008 PMC4067452 24685313

[7] Conic J, Lapinel N, Ali J, Boulmay B. Association between non-tuberculous mycobacterial infection and aerodigestive cancers: a case series highlighting different features, sequence and association. Respir Med Case Rep. 2022;40:101751. 10.1016/j.rmcr.2022.101751 PMC9574767 36262772

[8] Kusumoto T, Asakura T, Suzuki S, Okamori S, Namkoong H, Fujiwara H, et al. Development of lung cancer in patients with nontuberculous mycobacterial lung disease. Respir Investig. 2019;57:157–64. 10.1016/j.resinv.2018.11.004 30598398

[9] Lande L, Peterson DD, Gogoi R, Daum G, Stampler K, Kwait R, et al. Association between pulmonary mycobacterium avium complex infection and lung cancer. J Thorac Oncol. 2012;7:1345–51. 10.1097/JTO.0b013e31825abd49 22699888

[10] Beigi A, Naghib SM, Matini A, Tajabadi M, Mozafari MR. Lipid-based nanocarriers for targeted gene delivery in lung cancer therapy: exploring a novel therapeutic paradigm. Curr Gene Ther. 2025;25:92–112. 10.2174/0115665232292768240503050508 38778601

[11] Li CL, Ma XY, Yi P. The role of immunotherapy in lung cancer treatment: current strategies, future directions, and insights into metastasis and immune microenvironment. Curr Gene Ther. 2025;25:453–66. 10.2174/0115665232340926241105064739 39779552

[12] Qian XJ, Wang JW, Liu JB, Yu X. The mediating role of miR-451/ETV4/MMP13 signaling axis on epithelialmesenchymal transition in promoting non-small cell lung cancer progression. Curr Mol Pharmacol. 2024;17:e210723218988. 10.2174/1874467217666230721123554 37489792

[13] Uno S, Asakura T, Morimoto K, Yoshimura K, Uwamino Y, Nishimura T, et al. Comorbidities associated with nontuberculous mycobacterial disease in Japanese adults: a claims-data analysis. BMC Pulm Med. 2020;20:262. 10.1186/s12890-020-01304-6 PMC7547454 33036598

[14] Philley JV, Hertweck KL, Kannan A, Brown-Elliott BA, Wallace RJ Jr., Kurdowska A, et al. Sputum detection of predisposing genetic mutations in women with pulmonary nontuberculous mycobacterial disease. Sci Rep. 2018;8:11336. 10.1038/s41598-018-29471-x PMC6063893 30054559

[15] Cheol Seong S, Kim YY, Khang YH, Heon Park J, Kang HJ, Lee H, et al. Data resource profile: the National Health Information Database of the National Health Insurance Service in South Korea. Int J Epidemiol. 2017;46:799–800. 10.1093/ije/dyw253 PMC5837262 27794523

[16] Lee SW, Chang S, Chung E, Park Y, Kang YA. Effect of comorbidities on mortality in patients with nontuberculous mycobacterial infection in Korea: National Health Insurance Service-National Sample Cohort data. Sci Rep. 2024;14:22815. 10.1038/s41598-024-73768-z PMC11445539 39354035

[17] Yang B, Ryu J, Kim T, Jo YS, Kim Y, Park HY, et al. Impact of bronchiectasis on incident nontuberculous mycobacterial pulmonary disease: a 10-year national cohort study. Chest. 2021;159:1807–11. 10.1016/j.chest.2020.12.020 33373596

[18] Schneeweiss S, Rassen JA, Brown JS, Rothman KJ, Happe L, Arlett P, et al. Graphical depiction of longitudinal study designs in health care databases. Ann Intern Med. 2019;170:398–406. 10.7326/M18-3079 30856654

[19] Austin PC, Lee DS, Fine JP. Introduction to the analysis of survival data in the presence of competing risks. Circulation. 2016;133:601–9. 10.1161/CIRCULATIONAHA.115.017719 PMC4741409 26858290

[20] Benjamini Y, Hochberg Y. Controlling the false discovery rate - a practical and powerful approach to multiple testing. J R Stat Soc Ser B Stat Methodol. 1995;57:289–300.

[21] Kaneko H, Yano Y, Lee HH, Lee H, Okada A, Itoh H, et al. Medication-naive blood pressure and incident cancers: analysis of 2 nationwide population-based databases. Am J Hypertens. 2022;35:731–9. 10.1093/ajh/hpac054 35512273

[22] Suissa S. Immortal time bias in pharmaco-epidemiology. Am J Epidemiol. 2008;167:492–9. 10.1093/aje/kwm324 18056625

[23] Daley CL, Iaccarino JM, Lange C, Cambau E, Wallace RJ, Jr., Andrejak C, Bottger EC, Brozek J, Griffith DE, Guglielmetti L, et al: Treatment of nontuberculous mycobacterial pulmonary disease: an official ATS/ERS/ESCMID/IDSA clinical practice guideline. Eur Respir J. 2020;56:2000535. 10.1183/13993003.00535-2020. PMC8375621 32636299

[24] Lee G, Kim S, Chang S, Sohn H, Kang YA, Park Y. Epidemiological characteristics of nontuberculous mycobacterial pulmonary disease in South Korea: a meta-analysis of individual participant data. Tuberc Respir Dis (Seoul). 2024;87:386–97. 10.4046/trd.2023.0193 PMC11222092 38590075

[25] Hong SJ, Kim TJ, Lee JH, Park JS. Nontuberculous mycobacterial pulmonary disease mimicking lung cancer: clinicoradiologic features and diagnostic implications. Medicine (Baltimore). 2016;95:e3978. 10.1097/MD.0000000000003978 PMC4937910 27367996

[26] Ho M, Paruzzo L, Minehart J, Voruganti T, Devi P, Vogl DT, Cohen AD, Garfall AL, Waxman AJ, Kapur S, et al: Nontuberculous mycobacterial infections following teclistamab in multiple myeloma. Haematologica. 2025;110:1436–42. 10.3324/haematol.2024.286675. PMC12130765 39911104

[27] Lombardi A, Villa S, Castelli V, Bandera A, Gori A: T-Cell Exhaustion in Mycobacterium tuberculosis and Nontuberculous Mycobacteria Infection: Pathophysiology and Therapeutic Perspectives. Microorganisms. 2021;9:2460. 10.3390/microorganisms9122460. PMC8704935 34946062

[28] Fang L, Liu K, Liu C, Wang X, Ma W, Xu W, et al. Tumor accomplice: T cell exhaustion induced by chronic inflammation. Front Immunol. 2022;13:979116. 10.3389/fimmu.2022.979116 PMC9479340 36119037

[29] Kim HJ, Kwak N, Hong H, Kang N, Im Y, Jhun BW, et al. BACES score for predicting mortality in nontuberculous mycobacterial pulmonary disease. Am J Respir Crit Care Med. 2021;203:230–6. 10.1164/rccm.202004-1418OC 32721164

[30] Vedire Y, Kalvapudi S, Yendamuri S. Obesity and lung cancer-a narrative review. J Thorac Dis. 2023;15:2806–23. 10.21037/jtd-22-1835 PMC10267921 37324095

[31] Zou K, Sun P, Huang H, Zhuo H, Qie R, Xie Y, et al. Etiology of lung cancer: evidence from epidemiologic studies. J Natl Cancer Cent. 2022;2:216–25. 10.1016/j.jncc.2022.09.004 PMC11256564 39036545

[32] Yoo J, Lee Y, Park Y, Lee J, Choi JY, Lee H, et al. Update in association between lung cancer and air pollution. Tuberc Respir Dis (Seoul). 2025;88:228–36. 10.4046/trd.2024.0092 PMC12010724 39659117

[33] Millat MS, Hasan MM, Uddin MS, Salam MA, Aziz MA, Akhter I, et al. Inflammatory cytokines and specific factors influencing lung cancer progression. Cancer Pathog Ther. 2025;3:484–500. 10.1016/j.cpt.2025.04.002 PMC12573595 41179937

[34] Kim SH, Jhun BW, Jeong BH, Park HY, Kim H, Kwon OJ, et al. The higher incidence of COVID-19 in patients with non-tuberculous mycobacterial pulmonary disease: a single center experience in Korea. J Korean Med Sci. 2022;37:e250. 10.3346/jkms.2022.37.e250 PMC9424694 35971764

[35] Alkatout I, Biebl M, Momenimovahed Z, Giovannucci E, Hadavandsiri F, Salehiniya H, et al. Has COVID-19 affected cancer screening programs? A systematic review. Front Oncol. 2021;11:675038. 10.3389/fonc.2021.675038 PMC8165307 34079764

