# Supplementary material for: Cancer risk in patients with nontuberculous mycobacterial pulmonary disease: a nationwide population-based cohort study
Source: Respir Res. 2026 Jul 16;27:392. doi: 10.1186/s12931-026-03811-2 (PMC13656605; doi:10.1186/s12931-026-03811-2)
Supplement: Supplementary file 1 [file 12931_2026_3811_MOESM1_ESM.docx]

**Supplementary Materials**

**Contents**

**Supplementary Figure 1.** Competing-risk analysis of lung cancer risk in subgroups of patients with NTM-PD.

**Supplementary Table 1.** Sensitivity analyses excluding patients who developed cancer within different time frames.

**Supplementary Table 2.** Post-hoc analyses of cancer risk associated with macrolide-based treatment duration in patients with NTM-PD.

**Supplementary Table 3.** Post-hoc analyses of cancer risk by macrolide type and treatment duration in patients with NTM-PD.

**Supplementary Figure 1.** Competing-risk analysis of lung cancer risk in subgroups of patients with NTM-PD.

Forest plot showing subdistribution hazard ratios and 95% confidence intervals for lung cancer in patients with NTM-PD across different demographic and clinical subgroups. Analysis was performed using the Fine–Gray subdistribution hazard model instead of the cause-specific Cox proportional hazards model used in the main analysis, to account for competing mortality risk. *P*-values for interaction were calculated to assess effect modification across subgroups.

aHR, adjusted hazard ratio; BMI, body mass index; CI, confidence interval; HR, hazard ratio; NTM-PD, nontuberculous mycobacterial pulmonary disease

**Supplementary Table 1.** Sensitivity analyses excluding patients who developed cancer within different time frames.

|  | **<1 year** | | **<2 years** | | **<3 years** | |
| --- | --- | --- | --- | --- | --- | --- |
| **Cancer Type** | **aHR (95% CI)** | ***P*-value** | **aHR (95% CI)** | ***P*-value** | **aHR (95% CI)** | ***P*-value** |
| Overall cancer | 1.19 (1.13–1.25) | <.001 | 1.18 (1.11–1.24) | <.001 | 1.15 (1.08–1.22) | <.001 |
| Lung | 1.92 (1.74–2.13) | <.001 | 1.82 (1.62–2.05) | <.001 | 1.79 (1.56–2.05) | <.001 |
| Stomach | 0.89 (0.76–1.05) | .2 | 0.91 (0.76–1.09) | .3 | 0.88 (0.72–1.09) | .2 |
| Colorectum | 0.89 (0.77–1.03) | .1 | 0.82 (0.69–0.97) | .02 | 0.75 (0.61–0.93) | .007 |
| Liver | 1.20 (1.03–1.38) | .02 | 1.10 (0.93–1.31) | .3 | 1.15 (0.95–1.39) | .2 |
| Pancreas | 1.27 (1.09–1.48) | .002 | 1.19 (1.00–1.42) | .052 | 1.10 (0.90–1.36) | .4 |
| Ovary | 1.36 (1.06–1.74) | .01 | 1.58 (1.22–2.07) | <.001 | 1.71 (1.28–2.29) | <.001 |
| Multiple myeloma | 1.60 (1.11–2.31) | .01 | 1.95 (1.33–2.85) | <.001 | 1.91 (1.24–2.94) | .003 |

Sensitivity analyses were conducted using cause-specific Cox proportional hazards models by excluding patients who developed cancer within the first year (<1 year, corresponding to Sensitivity 2 in Table 2), first 2 years (<2 years), and first 3 years (<3 years) of follow-up to address potential detection bias.

aHR, adjusted hazard ratio; CI, confidence interval

**Supplementary Table 2.** Post-hoc analyses of cancer risk associated with macrolide-based treatment duration in patients with NTM-PD.

|  | **Macrolide ≥ 180 days** | | **Macrolide ≥ 365 days** | |
| --- | --- | --- | --- | --- |
| **Cancer Type** | **aHR (95% CI)** | ***P*-value** | **aHR (95% CI)** | ***P*-value** |
| Overall cancer | 1.08 (0.99–1.19) | .1 | 1.21 (1.09–1.34) | <.001 |
| Lung | 1.43 (1.19–1.71) | <.001 | 1.56 (1.27–1.91) | <.001 |
| Stomach | 1.52 (0.80–2.87) | .2 | 1.45 (0.70–3.01) | .32 |
| Colorectum | 0.82 (0.59–1.14) | .24 | 0.99 (0.69–1.43) | .97 |
| Liver | 1.11 (0.83–1.48) | .5 | 1.24 (0.90–1.72) | .19 |
| Pancreas | 1.16 (0.87–1.54) | .31 | 1.18 (0.86–1.63) | .31 |
| Ovary | 1.37 (1.02–1.83) | .04 | 1.30 (0.94–1.81) | .11 |
| Multiple myeloma | 0.70 (0.42–1.18) | .18 | 0.93 (0.53–1.61) | .79 |

Time-varying Cox proportional hazards models were used, adjusted for the same covariates as the primary analyses. To address immortal time bias, patients were classified as macrolide users only after reaching the predefined cumulative duration threshold (≥180 or ≥365 days).

aHR, adjusted hazard ratio; CI, confidence interval; NTM-PD, nontuberculous mycobacterial pulmonary disease.

**Supplementary Table 3.** Post-hoc analyses of cancer risk by macrolide type and treatment duration in patients with NTM-PD.

|  | **Clarithromycin ≥ 180 days** | | **Clarithromycin ≥ 365 days** | | **Azithromycin ≥ 180 days** | | **Azithromycin ≥ 365 days** | |
| --- | --- | --- | --- | --- | --- | --- | --- | --- |
| **Cancer Type** | **aHR (95% CI)** | ***P*-value** | **aHR (95% CI)** | ***P*-value** | **aHR (95% CI)** | ***P*-value** | **aHR (95% CI)** | ***P*-value** |
| Overall cancer | 1.03 (0.93–1.14) | .58 | 1.10 (0.98–1.24) | .1 | 1.18 (1.02–1.36) | .02 | 1.35 (1.14–1.59) | <.001 |
| Lung | 1.44 (1.18–1.74) | <.001 | 1.48 (1.18–1.85) | <.001 | 1.28 (0.97–1.70) | .09 | 1.49 (1.07–2.09) | <.001 |
| Stomach | 1.13 (0.56–2.28) | .74 | 1.14 (0.49–2.65) | .76 | 2.01 (0.88–4.60) | .1 | 2.38 (0.91–6.22) | .08 |
| Colorectum | 0.69 (0.47–1.02) | .06 | 0.76 (0.49–1.19) | .23 | 1.41 (0.91–2.20) | .13 | 1.81 (1.10–2.96) | .02 |
| Liver | 1.27 (0.94–1.73) | .13 | 1.34 (0.94–1.91) | .1 | 0.97 (0.60–1.56) | .89 | 0.99 (0.55–1.78) | .98 |
| Pancreas | 1.11 (0.82–1.51) | .5 | 1.08 (0.75–1.56) | .69 | 1.10 (0.71–1.71) | .68 | 1.33 (0.79–2.22) | .28 |
| Ovary | 1.12 (0.81–1.55) | .5 | 1.14 (0.78–1.66) | .51 | 1.63 (1.09–2.42) | .02 | 1.40 (0.84–2.35) | .2 |
| Multiple myeloma | 0.82 (0.46–1.45) | .49 | 1.06 (0.58–1.96) | .84 | 0.51 (0.20–1.27) | .15 | 0.65 (0.24–1.79) | .4 |

Time-varying Cox proportional hazards models were used, adjusted for the same covariates as the primary analyses. To address immortal time bias, patients were classified as clarithromycin or azithromycin users only after reaching the predefined cumulative duration threshold (≥180 or ≥365 days). Each macrolide type was analyzed separately.

aHR, adjusted hazard ratio; CI, confidence interval; NTM-PD, nontuberculous mycobacterial pulmonary disease.
